# Bovine endometrial MSC: mesenchymal to epithelial transition during luteolysis and tropism to implantation niche for immunomodulation

**DOI:** 10.1186/s13287-018-1129-1

**Published:** 2019-01-11

**Authors:** Alexandra Calle, Soraya López-Martín, Marta Monguió-Tortajada, Francesc Enric Borràs, María Yáñez-Mó, Miguel Ángel Ramírez

**Affiliations:** 1 Departamento de Reproducción Animal, Instituto Nacional de Investigación y Tecnología Agraria y Alimentaria (INIA), Avenida Puerta de Hierro 12, local 10, 28040 Madrid, Spain; 2 Departamento de Biología Molecular, UAM, Madrid, Spain; 3 REMAR Group and Nephrology Service, Germans Trias i Pujol Health Science Institute & University Hospital, UAB, Badalona, Spain; 4 Department of Cell Biology, Physiology and Immunology, Universitat Autònoma de Barcelona, Bellaterra, Spain; 5 CBM-SO, Instituto de Investigación Sanitaria Princesa (IIS-IP), Madrid, Spain

**Keywords:** Endometrial mesenchymal stem cells, Embryo implantation, Inflammation, Cell migration

## Abstract

**Background:**

The uterus is a histologically dynamic organ, and the mechanisms coordinating its regeneration during the oestrous cycle and implantation are poorly understood.

The aim of this study was to isolate, immortalize and characterize bovine endometrial mesenchymal stem cell (eMSC) lines from different oestrous cycle stages (embryo in the oviduct, embryo in the uterus or absence of embryo) and examine their migratory and immunomodulatory properties in an inflammatory or implantation-like environment, as well as possible changes in cell transdifferentiation.

**Methods:**

eMSCs were isolated and analysed in terms of morphological features, expression of cell surface and intracellular markers of pluripotency, inmunocytochemical analyses, alkaline phosphatase activity, proliferation and osteogenic or chondrogenic differentiation capacities, as well as their ability to migrate in response to inflammatory (TNF-α or IL-1β) or implantation (IFN-τ) cytokines and their immunomodulatory effect in the proliferation of T cells.

**Results:**

All eMSCs showed MSC properties such as adherence to plastic, high proliferative capacity, expression of CD44 and vimentin, undetectable expression of CD34 or MHCII, positivity for Pou5F1 and alkaline phosphatase activity. In the absence of an embryo, eMSC showed an apparent mesenchymal to epithelial transition state. eMSC during the entire oestrous cycle differentiated to osteogenic or chondrogenic lineages, showed the ability to suppress T cell proliferation and showed migratory capacity towards pro-inflammatory signal, while responded with a block in their migration to the embryo-derived pregnancy signal.

**Conclusion:**

This study describes for the first time the isolation, immortalization and characterization of bovine mesenchymal stem cell lines from different oestrous cycle stages, with a clear mesenchymal pattern and immunomodulatory properties. Our study also reports that the migratory capacity of the eMSC was increased towards an inflammatory niche but was reduced in response to the expression of implantation cytokine by the embryo. The combination of both signals (pro-inflammatory and implantation) would ensure the retention of eMSC in case of pregnancy, to ensure the immunomodulation necessary in the mother for embryo survival. In addition, in the absence of an embryo, eMSC showed an apparent mesenchymal to epithelial transition state.

## Background

In ruminants, embryo mortality is a key factor affecting fertility [1]. Approximately 50% of pregnancy loss occurs between blastocyst hatching and embryo attachment to endometrium in cattle [2]. Efficient communication between cells and tissues is paramount in many physiological processes, including embryo development. Preimplantational embryos of mammals develop within the female genital tract (oviduct and uterus) and their efficient communication conditions their development and survival. In ruminants, the bicornuate uterus is coated with the endometrium, the ultimate and unique biological layer facing the implanting embryo in normal early pregnancy, which drives the development of the embryonic disc and extra-embryonic tissues [3]. The endometrium displays two specific areas, namely the caruncles and the intercaruncular areas. The caruncles represent aglandular structures of limited size that are distributed over the endometrial surface. The intercaruncular areas are large and contain the endometrial glands, which produce histotroph, a collection of numerous and diverse factors including cytokines and growth factors [4].

During the window of implantation, after blastocyst attach onto the endometrium, both in human and mice, characterized by haemochorial implantation, the stromal cells at the implantation site begin to undergo decidualization, a process by which the fibroblast-like endometrial stromal cells differentiate into polygonal epithelial-like cells [5]. The differentiation of decidual cells is one of the earliest uterine adaptations to pregnancy in these species [6] and involves cell growth, change of shape, multinucleation and establishment of inter-cellular junctions [7]. Many genes and signal pathways are involved in the process of decidualization that is crucial for blastocyst implantation and maintenance of pregnancy. Ruminants and pigs have noninvasive implantation; however, they differ in the type of placentation. Pig conceptuses undergo epitheliochorial placentation in which luminal epithelium (LE) remains morphologically intact throughout pregnancy and the conceptus trophectoderm simply attaches to the apical LE surface without contacting uterine stromal cells [8]. In contrast, synepitheliochorial placentation in ruminants involves extensive erosion of the LE due to syncytium formation with trophectoderm binucleate cells. After day 19 of pregnancy, the conceptus tissue is apposed to, but does not penetrate, uterine stroma.

Johnson el al. reported that the uterine stroma of sheep undergoes a programme of differentiation similar to decidualization in invasive implanting species, whereas porcine stroma exhibits differentiation that is more limited than that in sheep, rodents or primates [9].

The uterus is a histologically dynamic organ, and the mechanisms coordinating its regeneration during the oestrous cycle and implantation are poorly understood. It has been proposed that bone marrow-derived cells contribute to uterine regeneration [10]. An alternative or additive mechanism of endometrial regeneration could be in situ cellular transdifferentiation, an example of which is mesenchymal to epithelial transition (MET). During MET, mesenchymal cells are re-programmed, thereby gradually losing mesenchymal cell characteristics while gaining epithelial cell traits [11]. MET and epithelial to mesenchymal transition (EMT) are fundamental processes that occur during the bovine embryo development and are also implicated in tumour metastasis [12]. Uchida et al. reported that the EMT might play an important role in human embryo implantation helping the embryo to penetrate through the endometrial epithelial cells into the endometrial stromal cell layer [13]. In addition, Zhang et al. suggested that MET might exist during decidualization, providing a stable developmental environment and an anchor point for embryos to invade into the uterus [14]. Recently, Patterson et al. reported that MET contributes to endometrial regeneration following natural and artificial decidualization by sesame oil injection into the uterine lumen in mouse [15], but the possibility that this cellular transdifferentiation also serves as a mechanism to regenerate adult endometrium in bovine has not been evaluated.

Although the presence of stem cells in the endometrium was first speculated in 1978 by Prianishnikov [16], it was not until 2004 that stem cells were identified and characterized for the first time in the endometrial tissue [17, 18]. The adult stem cells, mesenchymal stem cells (MSCs), unlike pluripotent embryonic stem cells, are multipotent and can produce a limited range of cell lineages depending on their location. MSCs are isolated from virtually every tissue, so that their stem cell properties are difficult to predict in an in vivo setting. The presence in the bovine endometrium of mesenchymal stem cells (eMSCs) has been already described [19–21]. Bovine eMSCs possess several MSC properties such as clonogenicity, ability to adhere to plastic, fibroblast morphology and in vitro differentiation capacity to adipocyte, chondrocyte and osteocyte [22]. Moreover, bovine eMSCs present high levels of CD29, CD44 and vimentin markers, low expression levels for CD34 and no MHC-II [21]. It is important to highlight that in some cases MSCs are able to divide but to a limited extent in vitro before entering replicative senescence. Between passages 7 and 12, MSCs increase their cell size and reduce the expression of certain pluripotency markers, leading to proliferative arrest [23].

MSCs have the ability to migrate to damaged and inflamed tissues where they proliferate and utilize their immunomodulatory properties as part of the regeneration process of the tissue itself [24–26]. The immune reaction has classically been functionally divided into Th-1 and Th-2 responses. Although, in human, numerous studies attempted to support pregnancy as a Th-2 or anti-inflammatory state [27], a similar number of studies supported its condition as a Th-1 or pro-inflammatory state [28]. A two-phase model was thereafter proposed by Mor et al., being the first and third trimesters characterized by a pro-inflammatory ambient, while the second trimester would represent an anti-inflammatory phase [29]. MSC can suppress or reduce the proliferation of T cells and also have a role in the regulation of Th-1/Th-2 balance shifting a Th-1 phenotype to a Th-2 [30]. MSC interactions with immune cells have been studied widely in both humans and rodents even in pigs [24], although this research has not yet been extended to MSC from the main veterinary species.

In ruminants, the major embryo-derived signal of pregnancy recognition has been suggested to be type I interferon [31]. The production of this interferon (interferon-tau; IFN-τ) is unique as it is secreted by trophectoderm cells during the elongation phase and secretion stops when trophectoderm cells appose to the endometrial luminal epithelium. IFN-τ is crucial for maternal recognition through the inhibition of endometrial prostaglandin-F2 alpha secretion, thus preventing luteolysis, an indispensable step for maintaining P4 luteal secretion [32].

Taking all this information into account, we have addressed the isolation, immortalization and characterization of eMSC from different phases of the bovine oestrous cycle in terms of their ability to migrate in vitro by agarose spot assay mediated by inflammatory cytokines (TNF-α or IL-1β) or the implantation cytokine (IFN-τ). We have also evaluated their possible immunomodulating function in a xenogeneic condition.

## Methods

### Isolation and culture of bovine endometrial mesenchymal stem cells (eMSCs)

Heifer uterus and ovaries were collected from routine slaughtered at the local abattoir. Oestrous cycle stages were determined by a macroscopic examination of the luteum corpus and ovarian morphology of the cycling heifers according to literature criteria [33] (see Table 1). The uterine horns ipsilateral to the luteum corpus were removed and washed with fresh tap water, and the uterine lumen was washed three times using a 20-ml syringe with Hank’s balanced salt solution (HBSS) supplemented with 500 U/ml penicillin, 500 mg/ml streptomycin and 0.1% bovine serum albumin (BSA) (Merck KGaA, Darmstadt, Germany) by flushing from the utero-tubal junction. To obtain bovine endometrial cells, a longitudinally cut of the uterine horn was performed with sterile scissors. The intercaruncular endometrium was carefully dissected from underlying tissues, cut in strips, minced using sterile scissors and incubated in 25-ml type II collagenase solution: HBSS supplemented 0.05% type II collagenase (Gibco by Life Technologies, Grand Island, NY, USA), 0.1% BSA (Merck KGaA, Darmstadt, Germany) and 30 mM CaCl2. The solution was homogenized and incubated during 1 h at 37 °C shaking gently every 10 min. The solution resulting from the digestion was filtered through a 70-μm cell strainer (SPL life science, Korea), and a volume of culture medium RPMI (Hyclone Laboratories, Utah, USA), supplemented with 10% foetal calf serum (PAA laboratories, Austria), 2% non-essential amino acids and antibiotics (100 U/ml penicillin, 100 mg/ml streptomycin), was added to block the action of collagenase and the obtained suspension centrifuged at 300 × *g* for 5 min. The resulting pellets were resuspended in culture medium and plated in 100-mm^2^ tissue culture dish (JetBiofil, Guangzhou, China) and incubated in an atmosphere of humidified air and 5% CO2 at 37 °C. Culture medium was changed every 48–72 h.

**Table 1:** eMSC isolation and immortalization efficiency

Oestrous cycle stage	Embryo location	Primary cultures	Immortalization efficiency	Immortalized eMSC	Oestrous stage
D2-D4 early luteal	Embryo in the oviduct	4	1 (25%)	1A	1
D4-D8 medium luteal	Embryo in the uterus with zona pellucid (ZP)	4	0 (0%)	–	2
D9-D16 late luteal	Embryo in the uterus w/o ZP	8	3 (37.5%)	3A, 3D, 3E	3
D17-D21 follicular phase	Regression of corpus luteum if not embryo	6	4 (66.7%)	4B, 4C, 4D, 4H	4

### eMSC immortalization

PA-317 LXSN-16E6E7 cells were acquired from the American Type Culture Collection (Manassas, VA). Virus was harvested from 16-h conditioned media of PA-317 cells that and filtered through a 0.45-mm low protein binding filter (Millipore, Bedford, MA) to remove cells and debris. Ten microlitres of viral supernatant was obtained from a confluent P100 dish. Viral supernatant was diluted 1:3 with serum-free DMEM/RPMI medium containing polybrene (4 mg/ml; Sigma). Primary cultures of bovine endometrial cells obtained from different cyclic stages which have been grown to 60% confluence in P100 culture dishes were overlaid with 3 ml of this mixture and incubated in an atmosphere of humidified air and 5% CO2 at 37 °C for 3 h. After 3–4 h, viral supernatant was removed; cells were washed twice with HBSS, and complete culture medium was added to dishes. After 48 h, cells having integrated the vector were selected by resistance to the neomycin analogue G418 300 μg/ml (Gibco of Life Technologies, Grand Island, NY, USA) in complete culture medium. Selection continued for 7 days, and surviving cells were subsequently cultured with complete culture medium containing 100 μg/ml G418 for general cell maintenance. The G418-resistant cell populations were subcultured for more than 30 passages, and immortalized cells were cryopreserved each passage.

### eMSC characterization by flow cytometry

Surface, cytoplasmic and nuclear cell antigens were analysed by flow cytometry using a Cell Lab Quanta SC system of Beckman Coulter. Cell cultures at 80–90% confluence were detached using 0.05% trypsin–EDTA (T/E) solution, collected and fixed with 4% paraformaldehyde for 10 min and subsequently washed twice with PBS. For the analysis of the expression of vimentin (clone LN-6, Sigma-Aldrich) and cytokeratin (Clone C-11, Sigma-Aldrich) (cytoplasmic proteins) and POU5F1 (rabbit polyclonal, Biorbit) (a nuclear protein), cell permeabilization was performed by incubation with 0.3–0.5% Triton X-100 for 10 min and washing with PBS. Nonspecific binding of antibodies was blocked with TNB-blocking solution during 30 min at 37 °C. Appropriated dilutions, provided by manufacturers, of primary antibodies against the markers commonly used to define mesenchymal stromal cells (MSCs): vimentin (clone LN-6, Sigma-Aldrich) and CD44 (clone IM7, Bio-rad), as positive markers; cytokeratin, CD34 (Rabbit polyclonal, Biorbyt), and MHCII (clone CVS20, Bio-Rad) as negative markers; and POU5F1 as pluripotency marker, were added to the cells and incubated overnight at 4 °C. Cells were then stained with the appropriated Alexa Fluor 488-conjugated secondary antibodies (Jackson InmunoResearch Laboratories, Baltimore Pike, West Grove, PA). Negative control samples were produced by the omission of the primary antibody. Analysis of the samples was performed with Cell Lab Quanta SC system of Beckman Coulter using Flow-Jo X SOFTWARE version 10.0.7r2.

### Alkaline phosphatase (AP) activity

eMSC lines at passages 10–15 were grown on 35-mm dishes (JetBiofil, Guangzhou, China) for 2 weeks. Cells were washed twice with PBS and fixed with a solution of 4% paraformaldehyde during 10 min at room temperature. Paraformaldehyde was aspirated, and the plates were washed twice with distilled water and covered with B solution (1 ml of Solution A (Fast Red 1 mg/ml), 1.6 μl of Napthol AS-mx phosphate and 40 μl TRIS HCl 1 M, pH 8.6) during 10–15 min at room temperature in the dark. Solution B was finally removed, and the cells were washed twice with PBS and covered with PBS to prevent drying. The colonies were studied for appearance of pink/red coloration indicating alkaline phosphatase activity. The stained colonies were imaged using a Motic SMZ-171 stereomicroscope coupled to a Moticam BTU8 digital camera using Motic Image Plus software version 2.0 (Motic China Group Co., LTD).

### Cell proliferation measurement

Different mesenchymal cell lines of passages 10–15 were seeded at 2 × 105 cells per 60-mm tissue culture plates (JetBiofil, Guangzhou, China). The culture medium was changed every 2 days. At each time point, a duplicate of plates was detached by tripsinization and counted using a Bürker counting chamber (Paul Marienfeld GmbH & Co. Lauda-Königshofen, Germany). Twenty microlitres of cell suspension was placed on both sides of the chamber and viewed using × 100 magnification under an inverted Nikon Diaphot phase contrast microscope coupled to a Jenoptik ProgRes CT1 digital camera. Images were taken using ProgRes capture pro software version 2.7 (Jenoptik Laser, Optic Systeme GmbH). A total of 5 × 1 mm^2^ squares per sample were analysed, and the number of cells per millilitre was determined according to number of cells in 1 ml = *N*/*Z* × dilution × 104, where *N* = the whole number of cells counted and *Z* = the number of counted squares. Cell population doubling time (PDT) was calculated using Roth V. 2006 Doubling Time Computing, available from http://www.doubling-time.com/compute.php.

### In vitro differentiation potential assay

eMSC lines at passages 10–15 were grown until 90% confluence on 24-well multidish (JetBiofil, Guangzhou, China). For osteogenic differentiation, The StemPro® Osteogenesis Differentiation Kit (Thermo Fisher Scientific, Meridian Road Rockford, IL, USA) was used according to the manufacturer’s instructions. Differentiating media was changed every 3–4 days for 21 days. Simultaneously, control cells were cultured in standard conditions. Cells were then fixed in 4% paraformaldehyde solution for 30 min. After fixation, cells were incubated for 2–3 min in 2% Alizarin Red S solution (pH 4.2) to visualize the calcium deposits.

For chondrogenic differentiation, The StemPro® Chondrogenesis Differentiation Kit (Thermo Fisher Scientific, Meridian Road Rockford, IL, USA) was used according to the manufacturer’s instructions. Differentiating media was changed every 2–3 days for 14 days. Simultaneously, control cells were cultured in standard conditions. Cells were then fixed in 4% paraformaldehyde solution for 30 min. After fixation, cells were incubated for 30 min with 1% Alcian Blue solution prepared in 0.1 N HCl. Blue staining was corresponding with proteoglycans synthetized by chondrocytes. Cells under differentiation conditions were photographed, using a Motic SMZ-171 stereomicroscope coupled to a Moticam BTU8 digital camera.

### Cell migration measurement: agarose spot assay

The cell migration measurement by agarose spot assay was carried out following the procedures of Calle et al. [34]. Briefly, PBS-0.5% agarose solution was heated on a water bath until boiling to facilitate complete dissolution. When the temperature cooled down to 40 °C, 90 μL of agarose solution was pipetted into a 1.5-ml Eppendorf tube containing 10 μL of PBS or PBS supplemented with TNF-α, IL-1β or IFN-τ for a final concentration of 6 nM [33]. Five-microlitre spots of agarose-containing PBS, TNF-α or IL-1β were pipetted onto six-well plates (JetBiofil, Guangzhou, China), 16 drops per well, 12 drops per MSC line, and allowed to cool for 15 min at 4 °C. At this point, cells that had been treated with 1 μg/ml C-Mitomycin overnight (Merck KGaA, Darmstadt, Germany) to avoid cellular duplication were plated onto spot-containing dishes in the presence of culture media and onto two dishes for mitomycin control, using Bürker counting chamber. Imaging was performed at 24 and 48 h using a Motic SMZ-171 stereomicroscope coupled to a Moticam BTU8 digital camera using Motic Image Plus software version 2.0 (Motic China Group Co., LTD). Motile cells penetrated the agarose spot. The longest straight distance from the border of the spot was analysed for each cell using ImageJ.

### In vitro xenostimulatory assays

To measure immunomodulatory properties of eMSCs, a co-culture of eMSCs and human T cells was performed. Human peripheral blood mononucleated cells (PBMCs) from peripheral blood of healthy donors were obtained by Ficoll Hypaque Plus (GE Healthcare) density centrifugation. T cells were then isolated from PBMCs using the Easy Sep Human T cell Enrichment Kit (Stem Cell Technologies) following the manufacturer’s instructions. Briefly, cells were diluted in a column buffer at a concentration of 5·10^7^ cells/ml, labelled with the tetrameric antibody complexes (TAC) recognizing human CD14, CD16, CD19, CD20, CD36, CD56, CD66b, CD123, glycophorin A and dextran-coated magnetic particles and separated using the EasySep magnet (StemCell Technologies). Enriched T cells were then washed and resuspended in PBS with an equal volume of CellTrace™ Violet (Thermo Fisher Scientific) for 10 min. After an overnight resting period, labelled T cells were washed twice with RPMI supplemented with 10% FBS and plated at 3·10^5^ cells/well in flat-bottomed well plates in which eMSC-1A or eMSC-4H had been previously seeded (50.000 cells/well) with RPMI medium. T cells had a viability of over 94% in all experiments performed and were stimulated with anti-CD2/CD3/CD28-coated microbeads (Pan T Cell Activation Kit; Miltenyi Biotech) or uncoated microbeads as a negative control in a 1:10 bead to T cell ratio. Xenoproliferation was determined after 4.5 days by measuring the CellTrace™ Violet loss by flow cytometry in a LSR Fortessa Analyzer (BD Biosciences).

### Statistical analysis

Statistical analysis was performed using GraphPad Prism 6 (GraphPad Software, La Jolla, CA, USA). One-way ANOVA for multiple comparisons by Fisher’s LSD tests were used for cell proliferation and doubling time. Two-way ANOVA for multiple comparisons by Fisher’s LSD tests were used for cell migration. Values are presented in mean ± standard error of the mean (SEM). Differences were regarded as significant when *p* < 0.05. Two-tailed Student’s *t* tests were used when two groups were compared for immunomodulatory assays. Values are expressed as mean ± standard error of the mean (SEM). Differences were considered to be significant when *p* < 0.05.

## Results

### MSC isolation and immortalization efficiency

eMSCs were isolated from heifer uterus that were ascribed to one of the four bovine oestrus stage categories (1–4) (Table 1) proposed by [33] based on the morphology of the active ipsilateral ovary to the uterine horn from which the cells were isolated. To avoid the risk of senescence by the maintenance of eMSC in vitro, isolated cells were immortalized using the retroviral vector LXSN-16E6E7. From a total of 22 primary cultures of endometrial stromal cells, eight cell lines were successfully immortalized (eMSC-1A, eMSC-3A, eMSC-3D, eMSC-3E, eMSC-4B, eMSC-4C, eMSC-4D, eMSC-4H) (Table 1).

### Morphological features

**Fig. 1:**
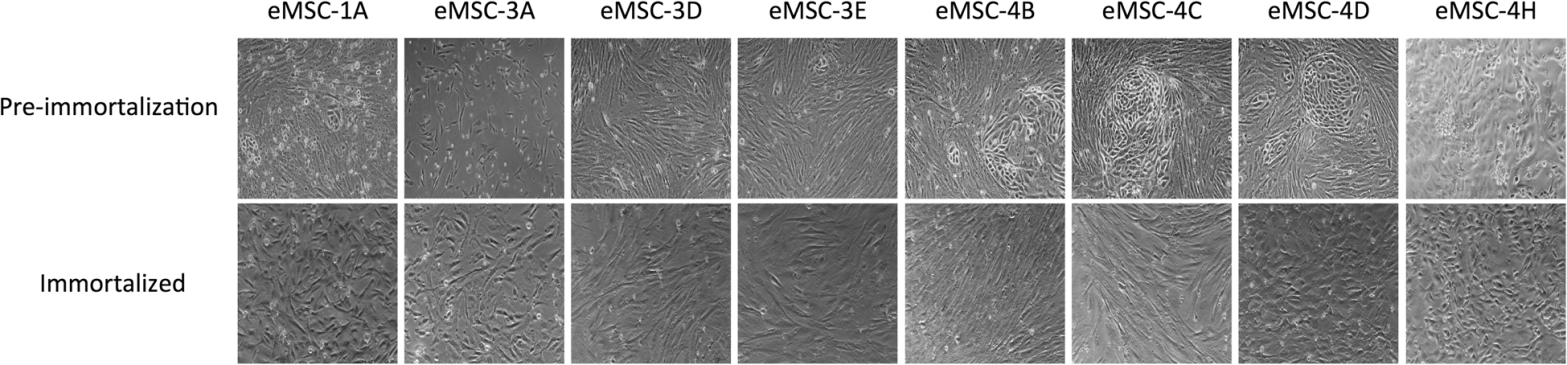
Morphology of MSCs. Pre-immortalized mesenchymal stem cell cultures at passage 0 (upper panels) and immortalized mesenchymal stem cell lines at passages 10–15 (lower panels). Phase-contrast images were acquired with × 100 magnification

Pre-immortalized mesenchymal stem cell cultures at passage 0 adhered to the plastic surface of culture dishes exhibiting a mixture of round, spindle or elongated shape morphology (Fig. 1—upper panels). However, after the first cell passage, the cells formed a more homogeneous population of fibroblast-like adherent cells, with the exception of eMSC-4D and eMSC-4H that showed an epithelial-like morphology remained constant before and after the immortalization even after more than 20 passages (Fig. 1—lower panels).

### Expression of cell surface, intracellular and pluripotent-specific markers

**Fig. 2:**
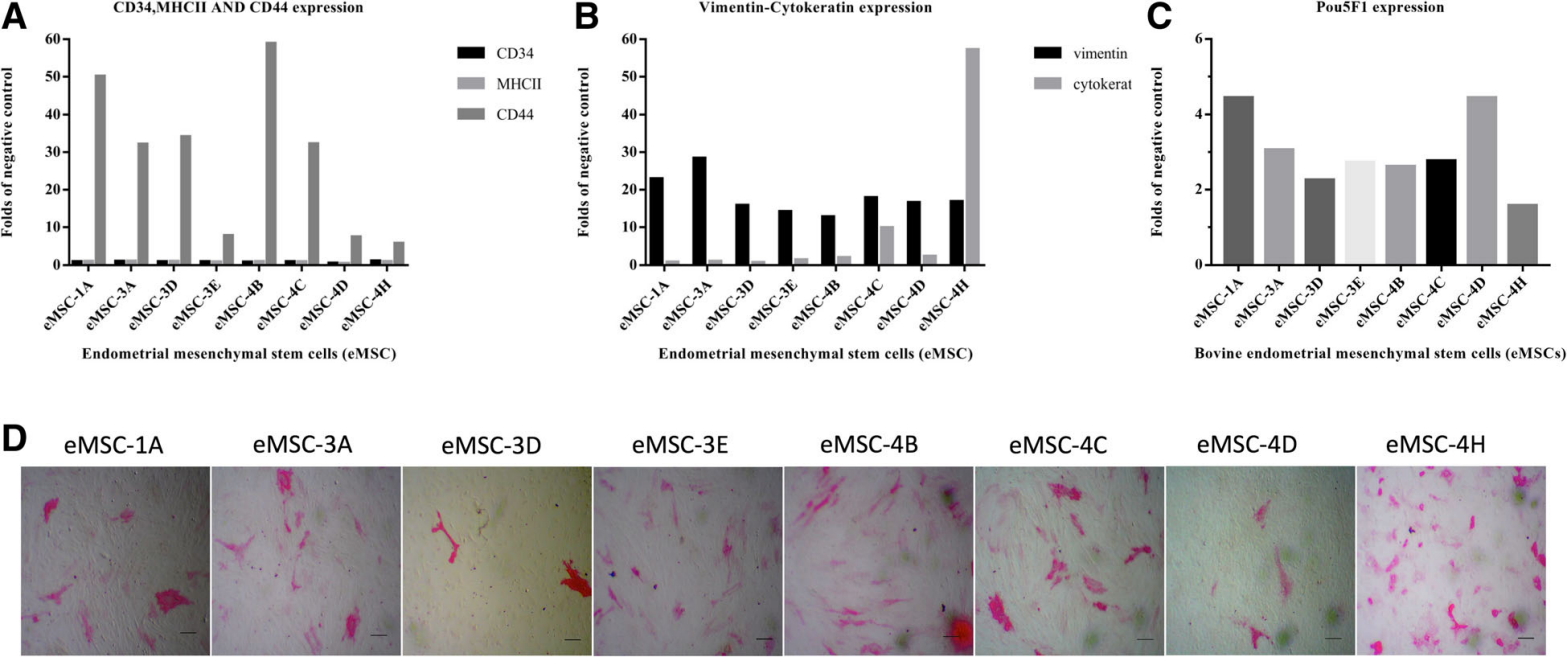
Expression of cell surface, intracellular and pluripotent specific markers and alkaline phosphatase activity. **a**–**c** Analysis by flow cytometry of the expression levels of cell surface markers CD34, CD44 and MHCII and intracellular markers cytokeratin, vimentin and POU5F1 in eMSC. Data correspond to the mean fluorescence intensity (folds of negative control) for each sample. **d** Analysis of AP activity: bright field images were obtained at × 50 magnifications, showing some red-stained cell groups after the action of alkaline phosphatase on Fast Red in the presence of Napthol AS-mx phosphate. Bars = 150 μm

Some characteristic MSC surface and intracellular markers were assessed by flow cytometry (Fig. 2a–c). All cell lines were positive for cell surface CD44 and cytoplasmic vimentin, both of them are characteristic markers of MSCs. Interestingly, cytokeratin, a typical cytoplasmic marker expressed by epithelium of ectoderm and endoderm, and commonly used as a negative marker of mesenchymal stem cells, was present in all stage 4 eMSC lines: strongly detected in eMSC-4H, clearly positive in eMSC-4C and slightly positive in eMSC-4B and eMSC-4D (Fig. 2b), correlating with the epithelial morphology of two of these cell lines. No expression of haematopoietic markers, such as MHCII or CD34, was found in any of the eMSC lines. Regarding pluripotency features, all eMSC lines, including those cell lines immortalized from the follicular phase and with epithelial morphology, were positive for the nuclear marker POU5F1 (Fig. 2c) and showed alkaline phosphatase activity (Fig. 2d).

### Proliferation capacity

**Fig. 3:**
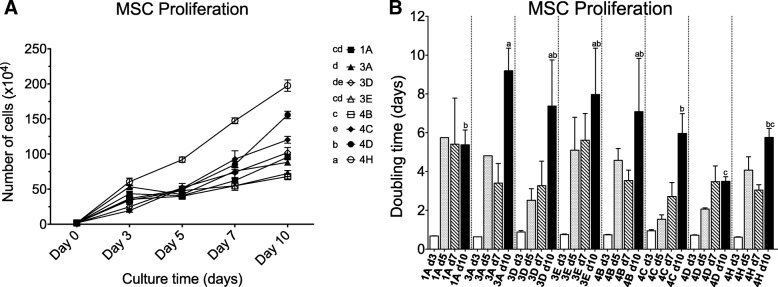
In vitro proliferation of mesenchymal stem cells. **a** Absolute number of cells/dish (mean ± SD) at indicated points. **b** Doubling time in days of each MSC line (mean ± SD). Different letters indicate significant differences (*p* < 0.05)

To analyse the cell proliferation capacity of eMSCs, the number of cells/dish was counted for each cell line at days 3, 5, 7 and 10, starting in all cases from an initial seeding of 2 × 10^5^ cells. As showed in Fig. 3a, the number of cells increased for all cell lines along the entire assay, demonstrating high proliferative capacity of these cells even at passages 10–15. eMSC-4H was the most proliferative line and at day 10 reached 19.74 × 10^5^ ± 2.13 × 10^5^ cells. Figure 3b shows the proliferation rate of MSC lines in terms of the population doubling time (PDT). Among eMSC lines, eMSC-4H and eMSC-4D showed on day 10 a significantly shorter PDT of 5.77 ± 0.47 and 3.50 ± 0.23 days, respectively (*p* < 0.05).

### In vitro differentiation of MSCs

**Fig. 4:**
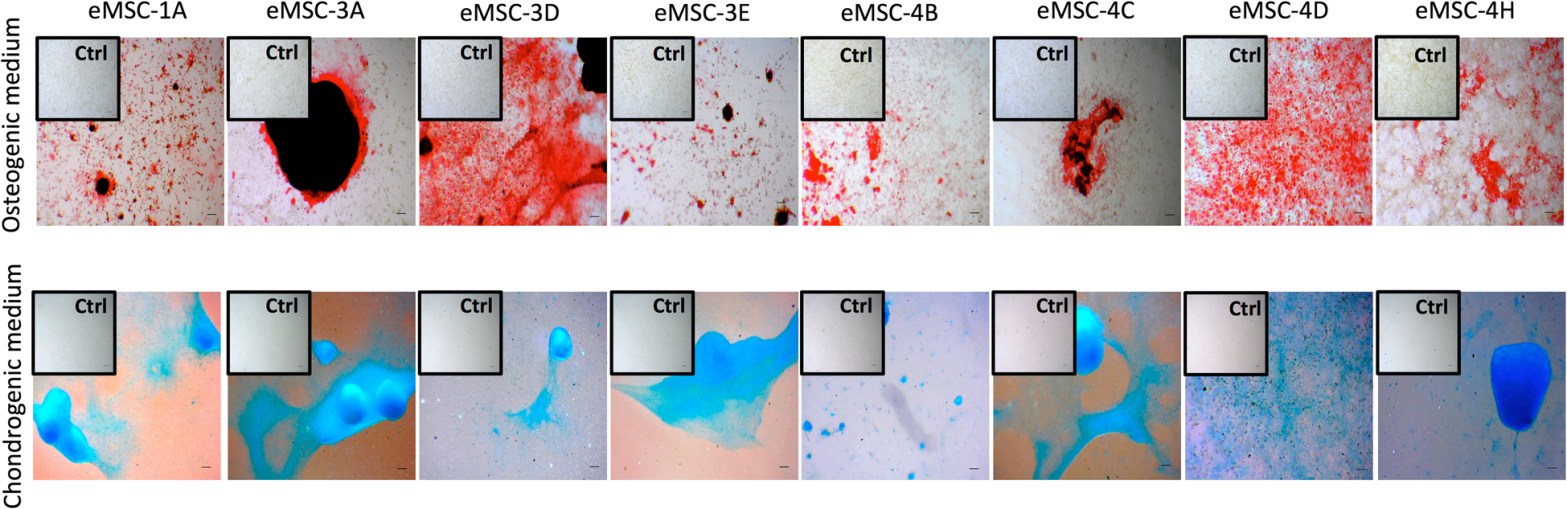
In vitro differentiation of eMSC to different lineages. Images show Alizarin Red S staining of calcium deposits in cells cultured in basal medium (control) or in osteogenic differentiation medium (top panel); Alcian blue staining of acidic proteoglycan in cells cultured in basal medium (control) or in chondrogenic differentiation medium (bottom panel). Bright field images were acquired with a × 3 magnification (bars = 150 μm)

As showed in Fig. 4, all eMSC lines cultured under osteogenic conditions presented a comparable amount of calcium deposits, indicating a high osteogenic differentiation potential of these lines. Cells cultured under chondrogenic conditions showed the presence of acidic proteoglycan that was demonstrated at monolayer cells by Alcian blue staining, presenting in most cases stained nodules typical from cartilaginous tissue.

All this characterization demonstrates that all immortalized lines complied with MSC features in terms of marker expression and differentiation capacities, even those obtained from the follicular phase that showed cytokeratin expression and epithelial morphology.

### Migratory response of MSC to inflammatory and implantational stimuli

**Fig. 5:**
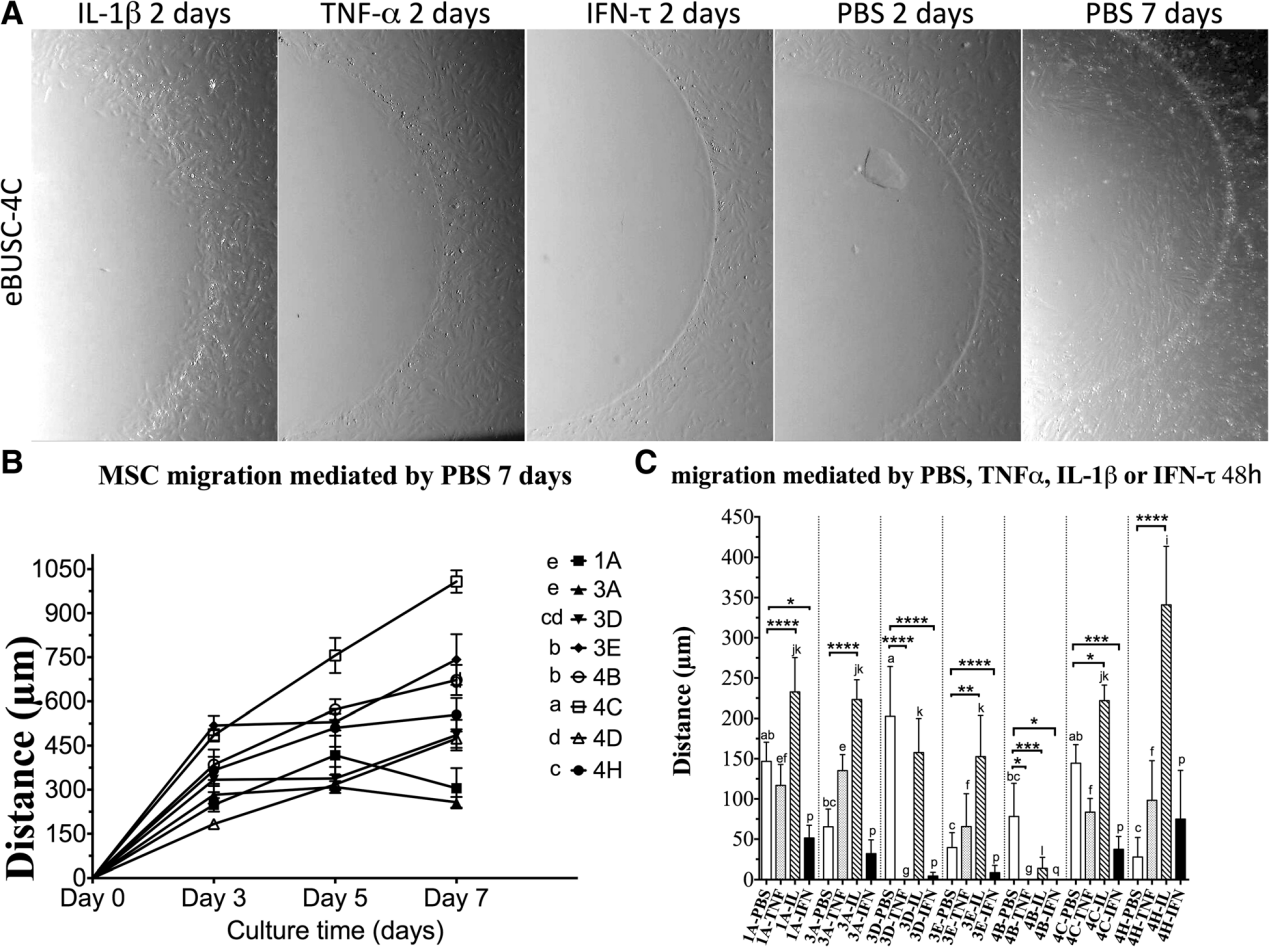
Migration analysis in an agarose spot assay. **A** Representative images of eMSC-4C migration assay into a PBS-, TNF-α-, IL-1β- or IFN-τ-containing agarose spot at the indicated times. Images were obtained in a light stereomicroscope at × 20 magnification. **b** and **c** Migratory response of MSC to inflammatory and implantational stimuli. **b** The distance migrated from the border of the PBS-agarose spot was measured in two independent experiments for all eMSC lines at the indicated times (mean ± SD). Different letters indicate significant differences: *p* < 0.005 for eMSC migration mediated by PBS at day 7 (a, b, c, d, e); **C** *p* < 0.05 for eMSC migration mediated by PBS (a, b, c), *p* < 0.005 for eMSC migration mediated by TNF-α (e, f, g) and *p* < 0.05 for eMSC migration mediated by IL-1β (i, j, k, l) and IFN-τ (p, q). *p < 0.05; ***p < 0.0005; ****p < 0.0001

Assessment of the invasive capacity of all eMSC lines was performed using the agarose spot assay [34], which allows the quantification of cell invasion by measuring their crawling underneath an agarose gel on a planar surface. All eMSC lines showed migration capacity into agarose spots without cytokine stimuli from day 2 (Fig. 5). In terms of migration without cytokine stimuli, eMSC-4C showed a significantly higher migration rate than the rest of eMSC lines with a net migrated distance of 1008.0 ± 38.26 μm on day 7 (Fig. 5B).

The inflammatory cytokine TNF-α did not cause an increase in the migration capacity of any of the eMSC lines studied compared to the control of PBS, but completely abrogated that of eMSC-3D and eMSC-4B. In contrast, the inflammatory cytokine IL-1β stimulated the migration capacity of cell lines eMSC-1A, eMSC-3A, eMSC-3E, eMSC-4C and eMSC-4H while caused a reduction in the cell migration rate of only the eMSC-4B line.

Regarding the preimplantation protein IFN-τ, it reduced cell motility of most cell lines (Fig. 5C).

### Immunomodulatory properties

**Fig. 6:**
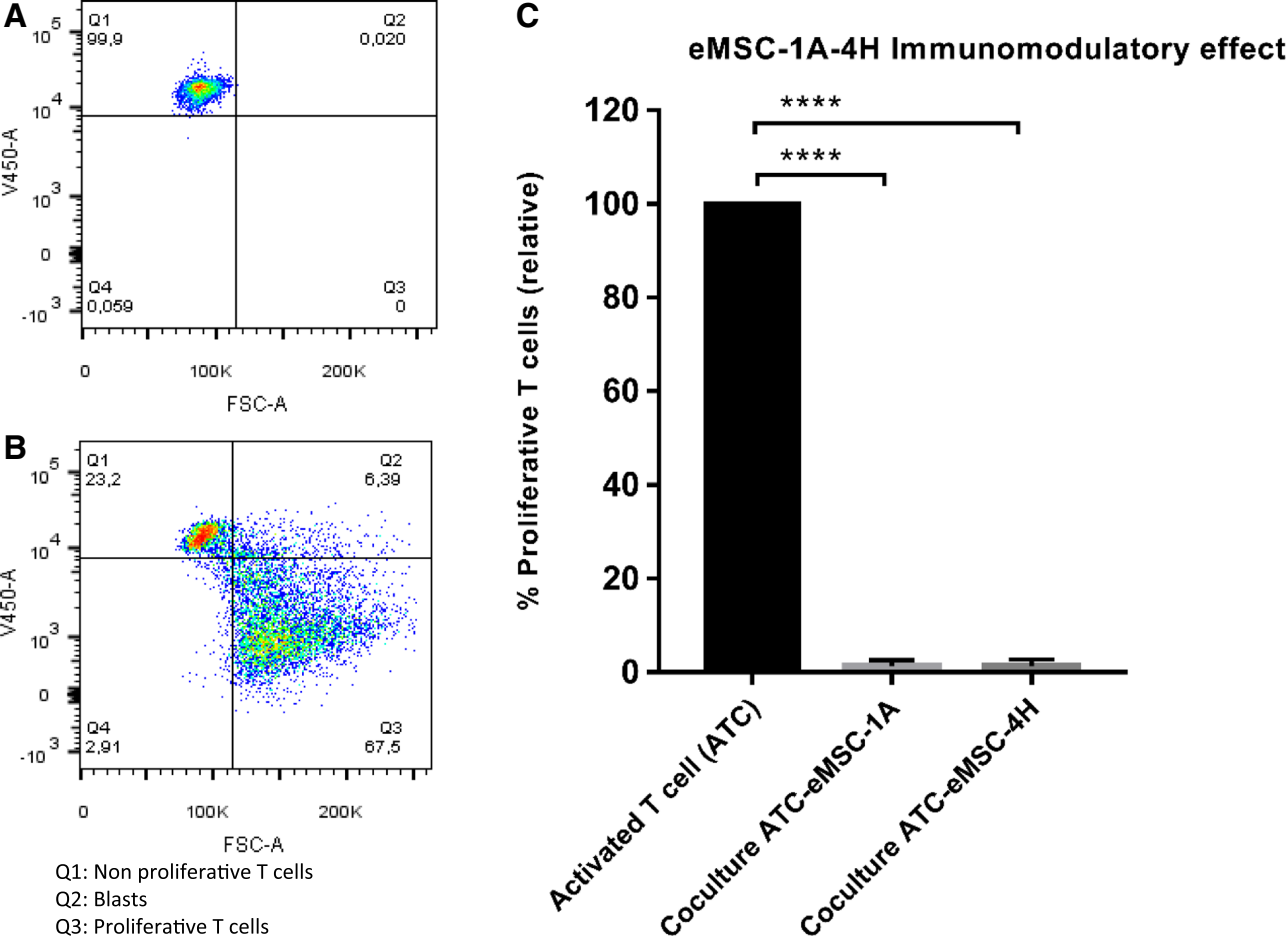
Measurement of T cell proliferation suppression capacity by eMSC. **a**, **b** Representative plots of non-stimulated and stimulated (anti-CD2/CD3/CD28-coated microbeads) human T cell control conditions (in the absence of MSCs). **c** Percentage of proliferating stimulated T cells in the presence of eMSC-1A or eMSC-4H (X:X T:eMSC ratio ± 0.54 and ± 0.63 respectively); proliferative T cell were analysed by Violet CellTrace fluorescence loss after a 4.5-day culture. Results are reported as percentage of proliferating T cells relative to the proliferation of activated T cells in the absence of eMSCs (positive control). ****p < 0.0001

Although all eMSC immortalized lines showed MSC differentiation capacities, those obtained from the follicular phase had unexpected features such as cytokeratin expression and epithelial morphology. Thus, we analysed the immunomodulatory potential of two representative lines: eMSC-1A with a pure mesenchymal pattern and eMSC-4H with a phenotype consistent with MET. Co-culture experiments with human T cells activated with anti-CD2/3/28 beads were established to analyse eMSC’s influence on polyclonal xenogeneic proliferation. The percentage of proliferating cells was determined by flow cytometry, quantifying FSC^high^Violet^low^ T cell blasts (Fig. 6a, b). An almost complete abrogation of activated human T cell proliferation was observed when these cells were co-cultured with either eMSC-1A (*p* < 0.0001) or eMSC-4H (*p* < 0.0001) (Fig. 6c).

## Discussion

We have successfully isolated, immortalized and characterized eMSC from different phases of the bovine oestrous cycle. The results of the present study clearly demonstrated that immortalized eMSC showed MSC properties such as adherence to plastic, high proliferative capacity until passages 10–15 and surface expression of CD44 and intracellular expression of vimentin, both MSC markers. Moreover, all eMSC lines were negative for haematopoietic specific markers CD34 and MHCII and positive for pluripotency-related marker Pou5F1, a nuclear protein that is well known for pluripotency maintenance and is expressed in bovine MSCs [20]. Numerous studies demonstrate that the ability to express alkaline phosphatase activity is also a pluripotency marker of stem cells [35]. In this report, immortalized eMSC lines from different phases of the bovine oestrous cycle showed positivity in AP activity.

All eMSC lines from early and late luteal phases showed a fibroblast-like morphology characteristic of MSC and expressed vimentin. Interestingly, eMSC lines obtained from the follicular phase (absence of an embryo) expressed cytokeratin and two of these lines showed a clear epithelial-like morphology. This observation could reflect a mesenchymal to epithelial transition (MET) of eMSC, a process in which mesenchymal cells are re-programmed, thereby gradually losing mesenchymal cell characteristics while gaining epithelial cell traits [11]. In both mouse and human, uterine regeneration was shown to occur both by bone marrow-derived cells [10] and also by MET of resident cells [14, 15]. Cousins et al. demonstrated that MET contributes to endometrial epithelium after progesterone withdrawal-induced tissue breakdown in a mouse model of menstruation [36]. This finding is consistent with Yu et al. observations showing that luteal hormone concentration and/or duration thresholds confer plasticity and reversibility of endometrial stromal phenotype via MET [37]. In bovine, progesterone produced by the corpus luteum is essential for the establishment and maintenance of pregnancy. Rapid regression of the cow’s corpus luteum is a key event in the bovine oestrous cycle as it is responsible for the precipitous decrease in the blood concentration of progesterone, the end of the cycle (follicular phase) [38]. Our eMSC lines established from the different phases of the oestrous cycle show a clear mesenchymal pattern in the early luteal phase (eMSC-1A, corresponding to the presence of the embryo in the oviduct) and late luteal phase (eMSC-3A, eMSC-3D and eMSC-3E, corresponding to the presence of the embryo in the uterus), while an apparent mesenchymal to epithelial transition state is observed in eMSC from the follicular phase (eMSC-4B, eMSC-4C and eMSC-4D, and eMSC-4H corresponding to the regression of the corpus luteum if there was no embryo) since they express cytokeratin and in some cases show an epithelial morphology. Considering that both the epithelium and the bovine stroma present oestrogen and progesterone receptors during the oestrous cycle [39], it would be interesting to analyse in future experiments the paracrine function performed by the eMSCs, thanks to the possible expression of hormone receptors during the oestrous cycle.

The ability to differentiate in vitro to mesodermal lineages has been demonstrated for eMSC isolated from the endometrium at the medium luteal phase [20] or medium and late luteal phase [21], but it was not known if the MSCs present in the uterus conserved such multipotency capacity during the entire oestrous cycle. Our data on osteogenic and chondrogenic induction with eMSC lines confirmed their multipotency, both in the early and late luteal phases as well as in the follicular phase.

An important characteristic of MSCs is their ability to migrate to damaged tissues or to inflammatory processes. All eMSC lines showed migration capacity without cytokine stimulation. There was not a clear difference in terms of non-stimulated migration between eMSC lines from different oestrus phases or with different morphologies (mesenchymal or epithelial). Although the eMSC lines obtained from the follicular phase (absence of an embryo) expressed cytokeratin and two of these lines showed clear epithelial type morphology, they also showed a high migration capacity and showed intracellular expression of vimentin. Vimentin is an important marker of mesenchymal to epithelial transition and a requisite regulator of mesenchymal cell migration. During MET, vimentin protein and mRNA expression are downregulated as cell motility decreases and cells adopt epithelial characteristics [40, 41]. Taking into account the above, MSCs show a cellular plasticity characteristic of MET that allows them to show characteristics of mesenchymal and epithelial cells. In all scenarios, either when the embryo is still in the oviduct (phase 1), or when the embryo without zona pellucida is in the uterus (phase 3), or in case of regression of the corpus luteum as a consequence of the absence of an embryo in the uterus (phase 4), most of the eMSCs present an active migration towards a pro-inflammatory niche (Th-1) characterized by the presence of IL-1β and described in the initial and final stages of human gestation [29]. In contrast, most of the eMSCs from the three scenarios described respond with a block in their migration to IFN-τ, the major embryo-derived pregnancy signal in bovine [31]. This is in clear contrast with the effect of this cytokine on bovine MSC derived from bone marrow, which are attracted by IFN-τ (Calle et al., under revision). The combination of both IL-1 and IFN-τ signals would ensure the retention of eMSC in case of pregnancy, as well as further recruitment of MSC from the bloodstream, to ensure the immunomodulation necessary in the mother for embryo survival.

eMSCs could act there as primarily suppressive effectors on Th1 cells by suppressing their proliferation, differentiation and activation [42, 43]. In accordance, both analysed lines; eMSC-1A, with a pure mesenchymal pattern and eMSC-4H with a phenotype consistent with MET, showed the ability to suppress T cell proliferation in vitro.

## Conclusion

**Fig. 7:**
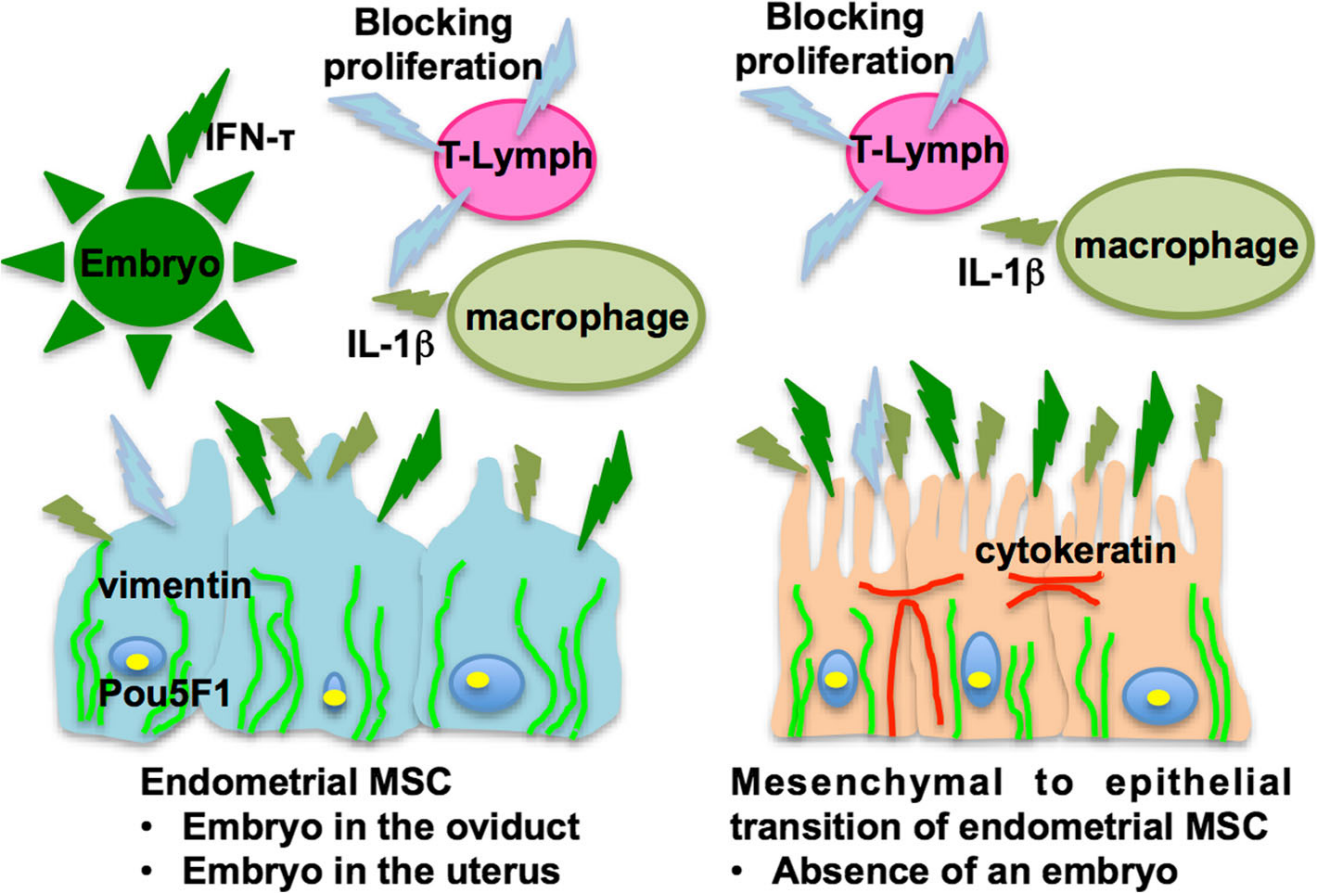
Model for bovine endometrial MSC: mesenchymal to epithelial transition during luteolysis and tropism to inflammation niche for immunomodulation

In sum, our results show that eMSC isolated and immortalized from the uterus shared common MSC features, although eMSCs isolated from the endometrium of the final phase of the oestrous cycle show a phenotype compatible with the mesenchymal-epithelium transition. Finally, the migratory capacity of eMSCs towards an inflammatory niche and their retention by interferon-τ secreted by the embryo would ensure the immunomodulatory ambient necessary for productive pregnancy (Fig. 7).

## Abbreviations

BSA: Bovine serum albumin
DMEM-LG: Dulbecco’s modified Eagle’s medium low glucose
eMSCs: Endometrial mesenchymal stem cells
EMT: Epithelial to mesenchymal transition
HBSS: Hank’s balanced salt solution
IFN-τ: Interferon-τ
IL-1β: Interleukin-1β
LE: Luminal epithelium
MET: Mesenchymal to epithelial transition
MHCII: Major histocompatibility complex II
PDT: Cell population doubling time
POU5F1: POU class 5 homeobox 1
T/E: Trypsin–EDTA
TAC: Tetrameric antibody complexes
TNF-α: Tumour necrosis factor-α
